# Image registration for *in situ* X-ray nano-imaging of a composite battery cathode with deformation

**DOI:** 10.1107/S1600577524000146

**Published:** 2024-02-01

**Authors:** Bo Su, Guannan Qian, Ruoyang Gao, Fen Tao, Ling Zhang, Guohao Du, Biao Deng, Piero Pianetta, Yijin Liu

**Affiliations:** a Shanghai Institute of Applied Physics, China Academy of Sciences, No. 2019 Jialuo Road, Shanghai 201800, People’s Republic of China; b University of Chinese Academy of Sciences, No. 19 Yuquan Road, Beijing 100084, People’s Republic of China; c Shanghai Synchrotron Radiation Facility, Shanghai Advanced Research Institute, Chinese Academy of Sciences, No. 239 Zhangheng Road, Shanghai 201204, People’s Republic of China; d Stanford Synchrotron Radiation Lightsource, SLAC National Accelerator Laboratory, 2575 Sand Hill Road, Menlo Park, CA 94025, USA; e Walker Department of Mechanical Engineering, The University of Texas at Austin, 204 E. Dean Keeton, Stop C2200, Austin, TX 78712-1591, USA; Tohoku University, Japan

**Keywords:** image registration, transmission X-ray microscopy, battery electrode deformation, chemical heterogeneity

## Abstract

The structural and chemical evolution of battery electrodes at the nanoscale plays an important role in affecting the cell performance. Nano-resolution X-ray microscopy has been demonstrated as a powerful technique for characterizing the evolution of battery electrodes under operating conditions with sensitivity to their morphology, compositional distribution and redox heterogeneity. In real-world batteries, the electrode could deform upon battery operation, causing challenges for the image registration which is necessary for several experimental modalities, *e.g.* XANES imaging. To address this challenge, this work develops a deep-learning-based method for automatic particle identification and tracking. This approach was not only able to facilitate image registration with good robustness but also allowed quantification of the degree of sample deformation. The effectiveness of the method was first demonstrated using synthetic datasets with known ground truth. The method was then applied to an experimental dataset collected on an operating lithium battery cell, revealing a high degree of intra- and interparticle chemical complexity in operating batteries.

## Abstract 2

A deep-learning-assisted image registration method is demonstrated for *in situ* X-ray nano-imaging of a composite battery cathode electrode with deformation. The method handles the challenges associated with electrode deformation by identifying and tracking isolated cathode particles separately. This approach could facilitate analysis of the correlation between intraparticle reaction heterogeneity and electrode deformation, which collectively affect the performance of real-world batteries.

## 1. Introduction

Lithium-ion batteries (LIBs) serve as key enablers in a variety of applications ranging from portable consumer electronics and electric vehicles to renewable energy storage systems. The broad adoption of LIBs has tremendous environmental and commercial impact and is indispensable in modern society. Real-world LIBs are characterized by structural and chemical complexities across a wide range of length and time scales. Various cell components including composite electrodes, electrolytes, separators and current collectors can change their respective morphological and chemical characteristics upon battery cycling, leading to a progressive performance degradation and even a risk of acute cell failure. It is therefore scientifically interesting and practically important to investigate the chemomechanical interplay and evolution in battery materials under operating conditions.

With exceptional capability in high-resolution and non-destructive imaging, nano-resolution X-ray microscopy has been identified as a powerful tool for battery research. Depending on the research goal, different imaging modalities have been developed. For example, nano-resolution X-ray radiography has been used to track the morphological change of S particles in an operating Li–S cell (Nelson *et al.*, 2012 ▸). The observed partial dissolution and passivation at the particle level indicate a potentially overlooked mechanism for Li–S cell degradation. Another good example is the combination of X-ray radiography with an X-ray energy scan to analyze spatially resolved signals of X-ray absorption near-edge structure (XANES). Xu and co-workers utilized this approach (also known as XANES imaging) to follow a single LiCoO_2_ cathode particle over more than 20 charge and discharge cycles with varying cycling rates (Xu *et al.*, 2017 ▸). With a XANES scan, sensitivity to the oxidation state of the targeted transition metal cations is achieved, indirectly revealing the evolution of lithium concentration and distribution in imaged active cathode particles. The nano-imaging results can also be extended to three dimensions by performing tomography, which has been successfully applied to several different battery systems (Wang, Chen-Wiegart & Wang, 2014*a* ▸,*b* ▸) to track their morphological complexities and dynamics, both of which can affect the cell behavior and life span.

The dynamic morphological evolution in battery materials introduces some technical challenges for X-ray nano-imaging. This is particularly the case when utilizing imaging modalities with a low temporal resolution, in which the sample’s structural deformation can cause significant imaging artifacts, jeopardizing downstream image analysis. For example, the execution of full-field XANES imaging involves acquisition of many transmission images as a function of X-ray energy (Malabet *et al.*, 2020 ▸; Pattammattel *et al.*, 2020 ▸; Zhang *et al.*, 2021 ▸). The state-of-the-art temporal resolution for this modality is roughly at the ten-minute level (Xie *et al.*, 2019 ▸). As a composite battery electrode could undergo non-rigid, non-linear and multidirectional deformation during electrochemical cycling, it could be very challenging to track all the structural features in an image accurately and very significant artifacts could be induced, hindering quantitative analysis of the XANES map. Additionally, practical limitations in the experiment, such as the instrument’s mechanical instability, or fluctuations in temperature, pressure and moisture, can also lead to image jitters, which need to be accounted for in the data reduction process.

Conventional image alignment methods have been utilized to address these challenges to a certain extent (Su *et al.*, 2022 ▸). They can be broadly classified into two groups. The first group evaluates the mutual information between the two images. Popular algorithms such as cross-correlation, phase correlation, fast normalized cross-correlation (FNCC) (Yoo & Han, 2009 ▸) and sum of squared differences (SSD) (Hisham *et al.*, 2015 ▸) all fall into the first category. The second group are known as feature-based alignment approaches. They employ feature detection and matching algorithms to align images. Examples of feature-based techniques include the scale-invariant feature transform (SIFT) (Burger & Burge, 2022 ▸), speeded-up robust feature (SURF) (Bay *et al.*, 2006 ▸) and optical flow (Nikitin *et al.*, 2021 ▸). These methods generally exhibit limitations in their ability to produce accurate results when the images are affected by significant noise, artifacts and sample deformations.

**Figure 1:**
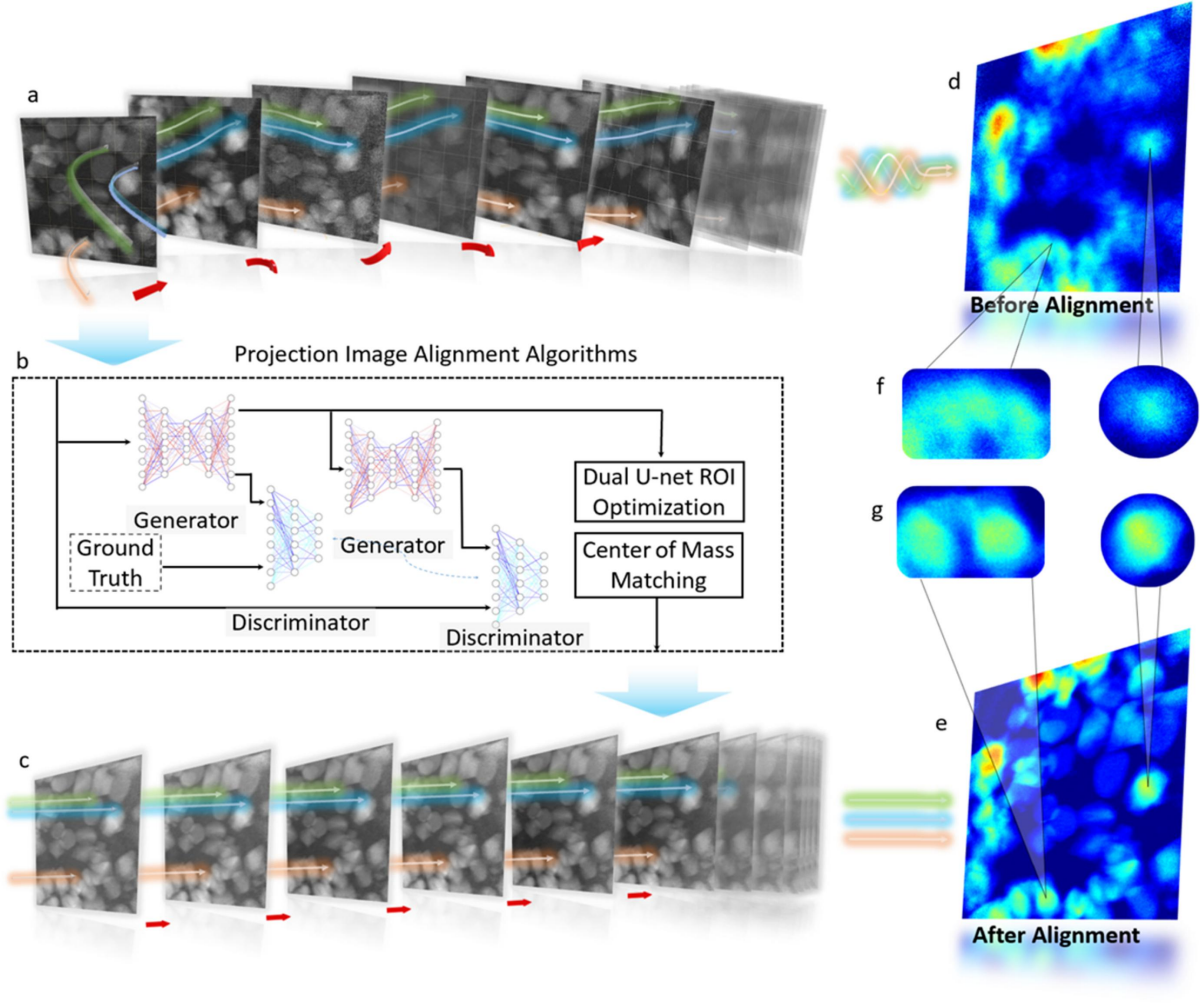
A schematic overview of the proposed algorithm. (*a*) A raw image stack of typical XANES imaging data with significant image jittering and deformation. (*b*) The deep-learning-based image alignment method, including the forward generator of *Cycle-GAN* and optimized *U-net*. (*c*) A schematic illustration of the alignment XANES image stack. (*d*)–(*g*) A comparison of the stacked image and enlarged regions of interest before [panels (*d*) and (*f*)] and after [panels (*e*) and (*g*)] applying our registration method.

In this work, a generative mask-based image alignment algorithm is proposed to address the above-mentioned challenges in X-ray nano-imaging of a composite battery cathode in an operating cell. Our method first detects isolated battery cathode particles in the image and then utilizes their respective center-of-mass coordinates to evaluate their non-rigid geometric distortion. This approach is particularly suitable for analyzing the nano-imaging data of a composite battery cathode because, while the electrode can undergo rather complicated deformation, the individual particles mostly remain structurally intact. The overall scheme of our approach is illustrated in Fig. 1 ▸. The raw nano-resolution XANES imaging data is fed into our model, and particles are detected and masked using *Cycle-GAN* (cycle generative adversarial network; Zhu *et al.*, 2017 ▸). This is followed by a few image denoising and background removal steps implemented with a *Dual U-net* network (Su *et al.*, 2022 ▸). The resulting sub-regions are used to determine the target’s center of mass and boundary, which are then used for particle registration and quantification of the electrode deformation.

We trained and validated our model using imaging data with single and multiple targets, meaning images with different numbers of isolated and detectable battery cathode particles. This model was then applied to real-world experimental data from the nano-imaging beamlines at both SSRF (Shanghai Synchrotron Radiation Facility, China) and SSRL (Stanford Synchrotron Radiation Lightsource, USA) with reasonable accuracy and robustness.

## 2. Method

### 2.1. Characteristics of XANES imaging datasets on deforming the battery material

Our group have developed and broadly applied the XANES imaging technique since the early 2010s. One of the most successful applications is in imaging battery cathode electrodes, which undergo both structural and chemical evolution as the battery is cycled. XANES imaging correlates the morphological changes and the local chemical evolutions, which are closely intertwined and collectively govern the battery performance.

In an ideal scenario, the sample should remain rigid throughout the energy scan. In practice, this approach faces technical challenges because the required energy scan takes around 10 min to accomplish and there could be considerable morphological evolution during the 10 min measurement. More specifically, the quality of the XANES imaging data reconstruction can be significantly affected by two factors: (i) imaging system imperfections and (ii) intrinsic sample deformation. The imaging system imperfections include instabilities of the sample stage and X-ray optics, as well as fluctuations of the X-ray source size, intensity and position. Meanwhile, sample deformation is closely associated with the intrinsic behavior of the battery. In addition, imaging noise plays a role in particle segmentation and contour detection. This is particularly the case for images acquired at lower X-ray energies, where the absorption is relatively weak and the contrast is poor. Therefore, we focus on detecting isolated particles and quantify their respective positions using their center of mass, which will be discussed in detail below.

### 2.2. Deep learning-based dynamic system alignment algorithm

**Figure 2:**
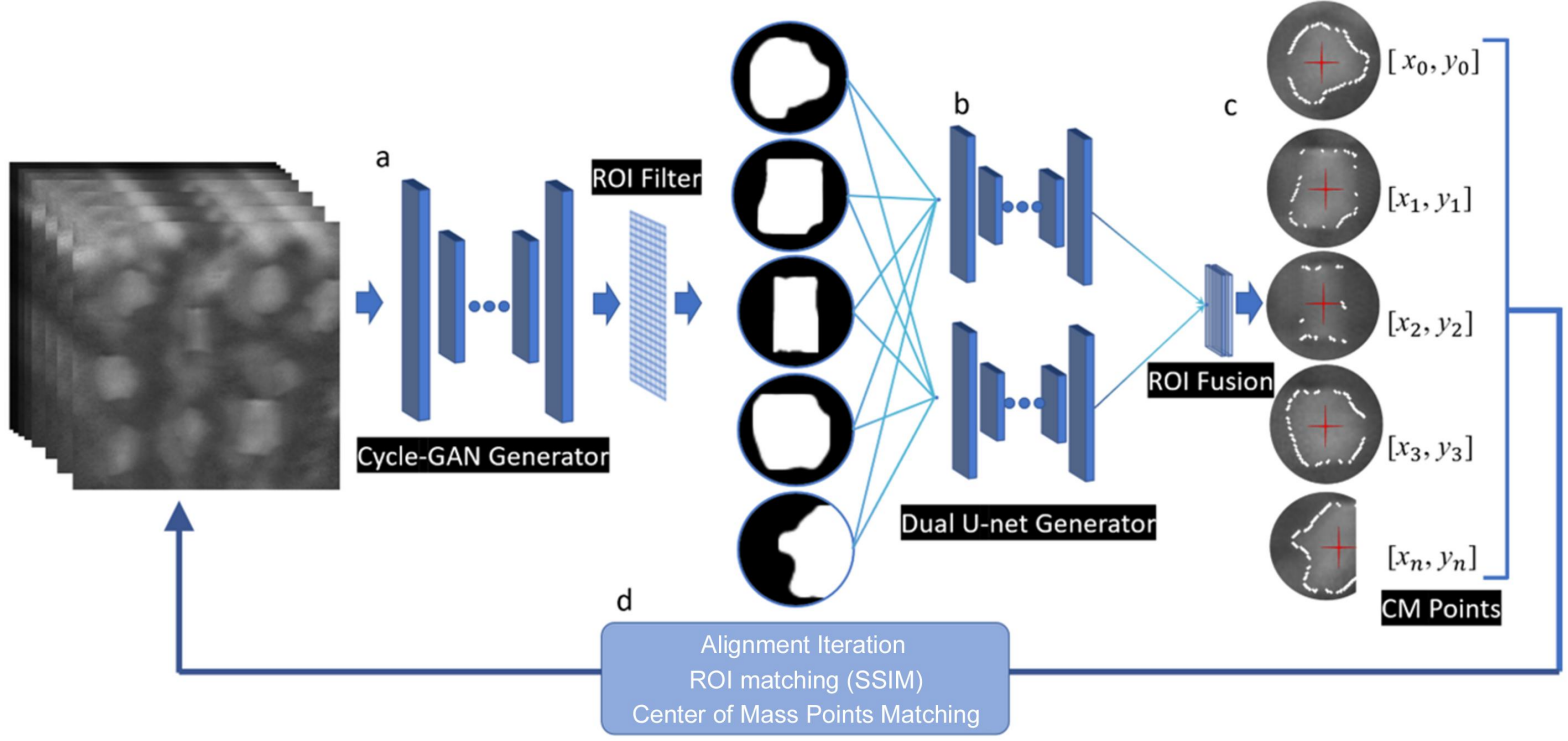
An illustration of the proposed algorithm. A *Cycle-GAN* generator is first used to identify isolated particles, leading to a set of ROI filters. A *Dual U-net* generator is then used to conduct registration of the ROIs separately. In the final step an ROI fusion approach is incorporated.

We divide the alignment task into a few steps and utilize different deep-learning models to address them respectively. We first implemented a model (*Cycle-GAN*) for the identification of multiple isolated particles in an image and subsequent definition of the respective regions of interest (ROIs). This step is repeated for images acquired at different energies and times. An ROI-matching step is implemented through evaluating the ROI similarities (SSIM) to ensure rough tracking of the identified particles. There are two reasons for dividing the whole image into several ROIs for independent processing downstream: (i) this approach could account for electrode deformation, which makes it nearly impossible to achieve global alignment with good accuracy, and (ii) the particle contour detection module (*Dual U-net*) works better with single target (particle) images. These ROIs are then processed for noise reduction and contour detection (*Dual U-net*) before we apply alignment using their respective centers of mass. The aligned ROIs are then subjected to XANES analysis using an established software package (Liu *et al.*, 2012 ▸). The whole process is illustrated in Fig. 2 ▸ and more details about each step are discussed in the sections below.

#### 2.2.1. Particle identification, ROI extraction and contour detection

The *Cycle-GAN* deep learning model is utilized in this research to detect multiple particle ROIs in order to refine their geometric information, such as particle contours, while mitigating complex background interference (changes in brightness or contrast and the occurrence of any interfering matter) and noise to yield high-quality results for a diverse dataset.

*Cycle-GAN* can be trained with unpaired datasets, resulting in a more robust model capable of scaling and transferring learning without the need for labeled data, allowing for the creation of faster and more accurate models with fewer data. The network structure consists of two generators and two discriminators.

Another deep-learning model used in this study is *Dual U-Net*, which was trained based on a reference model (Su *et al.*, 2022 ▸). *Dual U-Net* was used to optimize the ROI results generated from the *Cycle-GAN* model, specifically for denoising, background removal and single contour detection, which provided a single and extremely easy-to-describe target for ROI image alignment. The *Dual U-Net* network utilized two networks with the same structure, consisting of four down-sampling layers (step size of 2), four up-sampling layers (using an estimated interpolation with a step size of 2), 18 feature convolution layers (3 × 3 convolution kernels) and one fully connected layer.

#### 2.2.2. ROI matching

Consistent with the characteristics of XANES described in Section 2.1, a properly aligned XANES dataset should demonstrate a high degree of similarity between adjacent projections due to the energy tuning step employed during data acquisition. Accordingly, we employ the structural similarity index (SSIM) method (Sara *et al.*, 2019 ▸) to assess the similarity of every image pair within the segmented ROI for classifying the ROIs of different particles, and it can also be used to determine the effectiveness of the alignment technique based on its mean and variance. The similarity of paired projection images, and related variance and average, are calculated by the formula

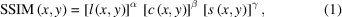

using the following parameters,

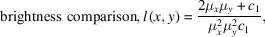




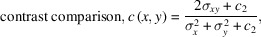




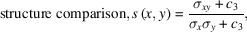

where α, γ, β, *c*_1_, *c*_2_ and *c*_3_ are constants, α = γ = β = 1, μ is the average value and σ is the standard deviation.

#### 2.2.3. Center-of-mass-based registration

Subsequent to the outcomes of the two deep learning models, the center-of-mass fluctuation technique (Aganj *et al.*, 2018 ▸) is utilized to perform the alignment of individual ROI images, as depicted in Figs. 2 ▸(*c*) and 2 ▸(*d*). Also, as a result of fluctuations in the scanning energy, particle edges in projection images may become blurred, leading to potential difficulties in XANES alignment. However, despite such changes, the distribution of pixel intensities within the particle itself remains uniform after processing by a deep learning model, as demonstrated by the recognition of the particles’ contours. This uniformity allows the center of mass to be calculated using the contours as a reliable feature for alignment. Using information on the particle’s contour, the zeroth- and first-order moments of its geometry (Joseph-Rivlin *et al.*, 2019 ▸) can be used to calculate the center of mass. The zeroth-order moment is the summation of all non-zero pixels within the contour and can be expressed mathematically as

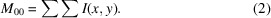

The first-order moment is the sum of the pixel coordinates multiplied by their intensity values, divided by the zeroth-order moment. This moment can be mathematically represented as

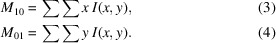

Using these moments, the horizontal and vertical coordinates of the center of mass can be obtained as

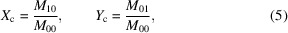

where the following parameters are used: *I*(*x*, *y*) is the pixel value at position (*x*, *y*) in the image, representing the pixel intensity at that location; *M*_00_ is the zeroth-order moment of the image, which is the sum of all pixel intensity values in the image; *M*_10_ is the first-order moment of the image, which is the average of the product of pixel intensity values and their *x* coordinates, representing the average position of the particle along the *x* axis; *M*_01_ is the first-order moment of the image, which is the average of the product of pixel intensity values and their *y* coordinates, representing the average position of the particle along the *y* axis; *X*_c_ is the horizontal coordinate of the center of mass of the particle; and *Y*_c_ is the vertical coordinate of the center of mass of the particle.

## 3. Experimental methods

### 3.1. Battery electrode and cell preparation

A coin cell with an X-ray-transparent Kapton window was assembled for this experiment. The cathode electrode was made of a monolayer of single-crystalline LiNi_0.6_Co_0.2_Mn_0.2_O_2_ (NCM_622_) particles. The electrode was vacuum dried at 85°C for 12 h. Electrolyte [1.2 *M* LiPF6, EC (ethylene carbonate): DMC (dimethyl carbonate): DEC (diethyl carbonate) = 1:1:1 (by weight) and 1.5 wt% VC (vinylene carbonate) additive] was filled in an argon-filled glovebox (O_2_/H_2_O < 0.1 p.p.m.). Battery charging was performed using a battery cycler (Biologic SP300). The cell was first cycled twice between 2.7 V and 4.25 V as the formation cycles. After that, the cell was charged at rate of 0.1 C while the X-ray characterization was conducted.

### 3.2. TXM configuration and XANES imaging protocol

The experimental data utilized in this work were acquired on beamline 6-2C at the Stanford Synchrotron Radiation Lightsource and on the 18B 3D nano-imaging experimental beamline station at the Shanghai Synchrotron Radiation Light Source (Tao *et al.*, 2023 ▸). The imaging procedure employed a combination of full-field nano-resolution imaging using a transmission X-ray microscope (TXM) and XANES. The TXM setup utilizes a capillary ellipsoidal focusing mirror (Mono Capillary) as the condenser, which is followed by a pinhole, a sample stage (*x*, *y*, *z* and rotation), a zone-plate objective lens and an area imaging detector (CCDs). The illuminated field of view (FOV) is approximately 20 µm × 20 µm and the energy ranges from 5 keV to 14 keV, which covers the absorption *K*-edges of several transition metal elements relevant to battery applications. The energy resolution (Δ*E*/*E*) is about 2 × 10^−4^, which is sufficient for XANES imaging experiments. In addition to sample deformation, observed image misalignments are also due to imperfections of the imaging system, including the limited stability of the synchrotron source and hardware along the beamline and in the endstation. During the experiment, the sample was repeatedly moved in and out of the beam to acquire images with and without (*i.e.* background) the sample in position.

The full dataset contains 6435 projection images. We used 286 images for training our models, and the remaining images were used for validation, and for characterizing the physicochemical response of the battery electrode during charging cycles. For each XANES scan, we cover the Ni *K*-edge with 143 energy points, providing sufficient energy resolution to resolve the difference in local Ni oxidation states. The entire image stack includes 54 XANES scans for revealing the chemical dynamics of the particles upon charging at a slow rate. While each of the raw images has 1000×1000 pixels, we effectively reduce the field of view to 460×460 pixels to avoid image artifacts at the edge of the FOV due to insufficient illumination.

The computational workstation utilized in this study was implemented using Python 3.8 and compiled with PyTorch 1.8.1 and CUDA 10.2. The projections alignment process was executed with an Intel Xeon W-2245 central processing unit (CPU) and an Nvidia Quadro 5000 graphics processing unit (GPU) with 256 GB RAM.

## 4. Results and discussion

**Figure 3:**
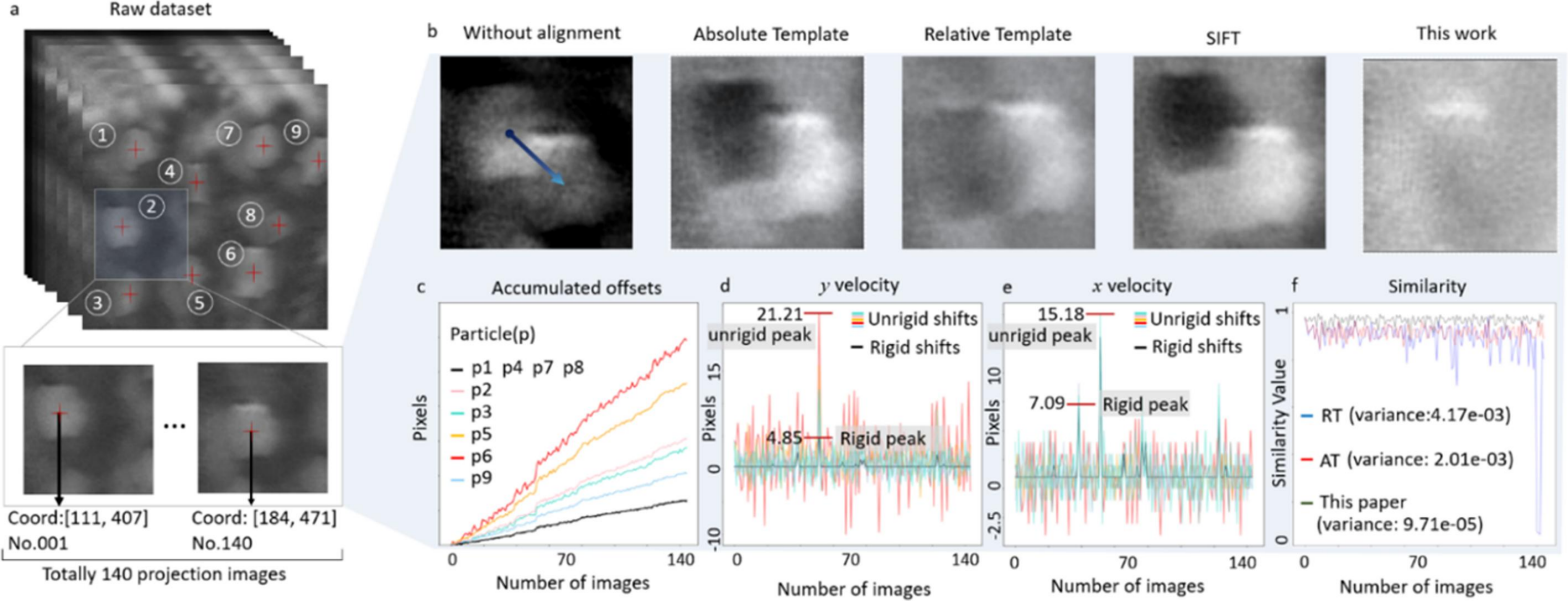
Analysis of the image alignment results of a synthetic dataset. (*a*) The image stack with enlarged ROI to illustrate the image distortion. (*b*) A comparison of the registration results from several different algorithms using differential maps. (*c*)–(*f*) Quantitative evaluation of the alignment results.

Here we compare our image alignment method against other conventional approaches, *e.g.* the absolute template method, the relative template method and the SIFT method. In Fig. 3 ▸ we present the results of different alignment algorithms on registering a synthetic XANES image stack that contains 143 projection images. This synthetic dataset is designed for evaluating the comparative effectiveness of these algorithms with various distortion modes, including global rigid offsets, random relative offsets, image background variation and noise fluctuation. We visualize the full synthetic dataset in Video S1 of the supporting information.

In this dataset, our model identified nine separated particles and we focus on comparing the alignment results of these particles with different algorithms. To visualize the alignment quality achieved by different algorithms, we subtract the last image from the first image of the aligned image stack and evaluate the residual values in a differential map. For instance, in the ROI containing particle 2 [Fig. 3 ▸(*b*)], all three conventional methods (absolute template alignment, relative template alignment, SIFT alignment) demonstrate quite significant limitations, as shown by the shadowing features in the differential maps. This is caused by non-rigid sample deformation and the limited image quality, both of which were purposely introduced into the synthetic dataset. The optical flow method was considered but the alignment failed, because the energy used in the acquisition of XANES varies continuously, resulting in continuous dynamic changes in brightness, contrast and background, which markedly curtail the effectiveness of this method for image alignment (Barron *et al.*, 1994 ▸; Sun *et al.*, 2010 ▸). Our approach, on the other hand, demonstrates an improved performance, with the ability to eliminate accurately all edges and internal regions of particle 2 in the differential map. Our method retains features that exhibit different pixel values that originate from the different scan energies, which is critical for XANES spectrum reconstruction. Thanks to the use of this synthetic dataset, we can compare the alignment results against the ground truth. We quantified the interparticle offset resulting from our alignment method using similarity and variance distribution, which enabled us to assess the accuracy of the method. As illustrated in Fig. 3 ▸(*f*), for this particle the proposed method exhibits a significantly suppressed variance and an averaged similarity accuracy of 98.32%. In the supporting information we present the results of the differential maps for several other particles. These results are similar to what we have shown in Fig. 3 ▸, highlighting the robustness of our approach.

In addition, by tracing these particles independently, we can monitor the displacement of individual particles, revealing electrode-scale non-rigid deformation. We combine contour detecting techniques and the center-of-mass calculation to track the identified particles independently. The cumulative displacement of each individual particle and the horizontal and vertical components of the particle motion velocity in consecutive image frames are plotted in Figs. 3 ▸(*c*), 3 ▸(*d*) and 3 ▸(*e*), respectively. This information is not only essential to registering each individual battery particle to reconstruct their respective XANES signals but also provides some insight into the dynamic deformation behavior at the electrode level. Fig. 3 ▸(*c*) illustrates that the slowest accumulation rate (represented by the black line) corresponds to particles with only global rigid displacements, which could caused by instability of the microscope hardware rather than deformation of the sample. All the other colored lines represent particles with non-rigid displacements. The smooth black lines in Figs. 3 ▸(*e*) and 3 ▸(*f*) represent the horizontal and vertical velocity components of particles with only global rigid displacements, while all other colored lines correspond to particles with non-rigid dynamic random displacements. The maximum displacement amplitude for non-rigid motion is significantly higher than that of rigid motion, exhibiting a peak 4.37 times greater in the vertical direction and 2.14 times greater in the horizontal direction. It is evident that non-rigid displacements accumulate more significantly than global rigid displacements, and the other velocity components are significantly greater than the global ones. Therefore, the variance of the global displacement squared and displacement velocity for all particles is substantial, which severely affects the effectiveness of the traditional alignment algorithms that rely on global grayscale information (template) and multiple feature recognition (SIFT) for overall integrated displacement correction. Furthermore, the computational efficiency is very high, as exemplified by processing of a dataset including 100 XANES projection images. Each image, measuring 460×460 pixels and containing nine particles, undergoes computations for offset velocity, acceleration and distance for each particle, completing the task in less than 10 s. This results in significant impacts not only on the alignment of particles with non-rigid displacements but also on the alignment of particles with only rigid displacements in the scenarios that both cases coexist.

**Figure 4:**
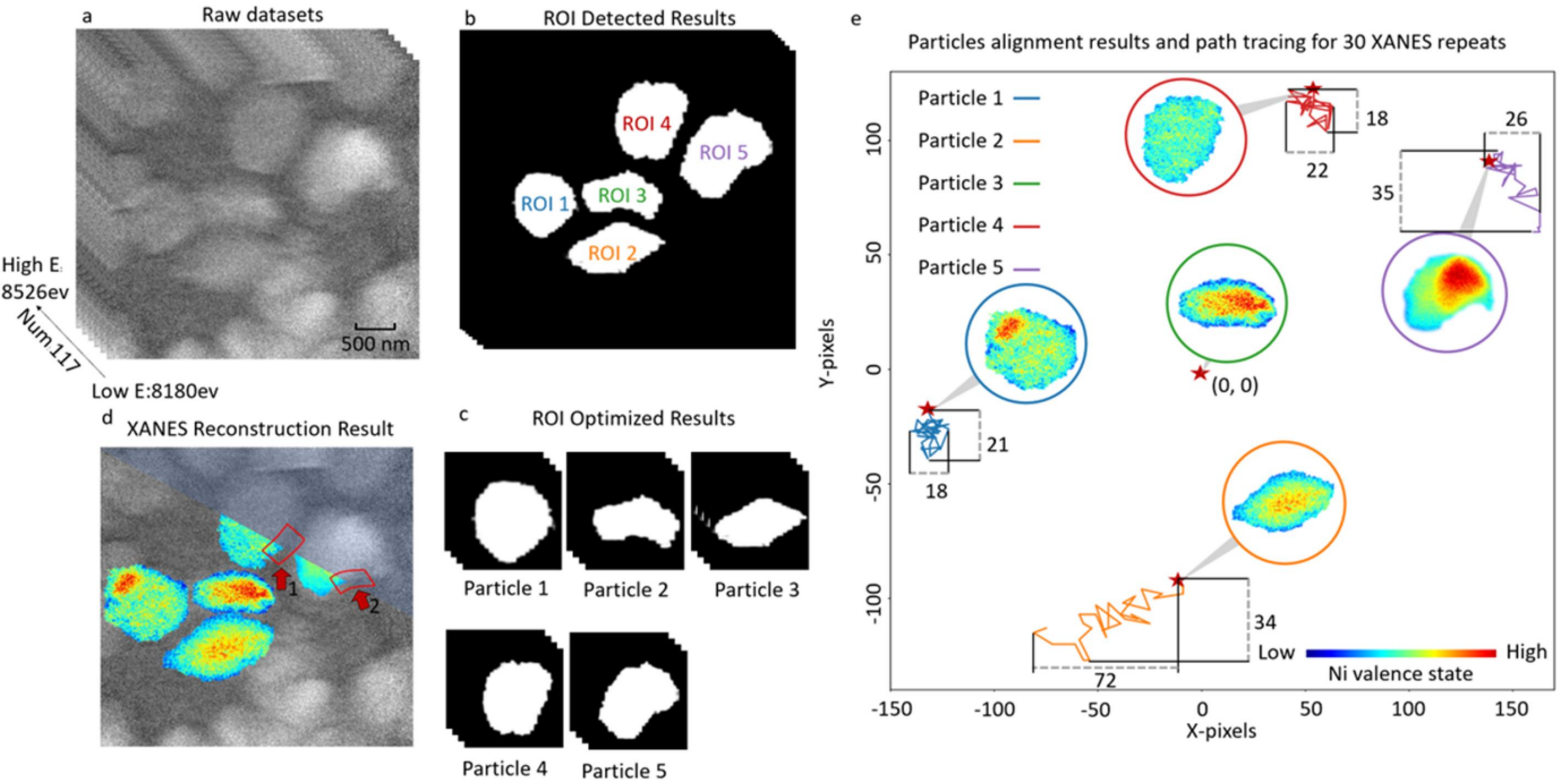
Alignment and analysis of *operando* XANES imaging of an NCM battery cathode upon charging. (*a*) Raw TXM images. (*b*) The results of ROI detection by *Cycle-GAN*. (*c*) The results of particle segmentation. (*d*) The results of XANES map reconstruction for the identified particles. (*e*) The Ni oxidation maps over the segmented and tracked particles at the end of the charging process, and their relative spatial offsets.

The proposed algorithm is further applied to rectify mis­alignments in TXM-XANES data of an NCM cathode electrode that is being actively charged. The details of the electrode and cell configuration, the instrument setup, and the experimental protocol can be found in the *Experimental methods* [Sec sec3] section. The raw data, presented in Fig. 4 ▸(*a*) and in Video S2 of the supporting information, exhibit noticeable global rigid jitters, non-rigid deformations, and varying image background and noise. Through our method, several isolated particles are individually identified and independently aligned with their boundaries optimized, as shown in Figs. 4 ▸(*b*) and 4 ▸(*c*). Subsequently, the aligned image stacks for these particles were subjected to XANES reconstruction, and the reconstructed Ni oxidation maps are shown in Fig. 4 ▸(*d*).

Although redox heterogeneity at the particle level is a broadly reported phenomenon in battery cathodes, the pattern of the XANES map can demonstrate very different features and the mechanisms are not well understood. For example, Kuppan and co-workers showed a chemical gradient in LiMn_1.5_Ni_0.5_O_4_ cathode particles that indicates chemical onset on one side and propagates through the particle in quite a complicated pattern (Kuppan *et al.*, 2017 ▸), which is attributed to an imperfect particle morphology with crystal truncation on its corners. Wang and co-workers demonstrated a 3D shrinking-core pattern in an LiFePO_4_ particle (Wang *et al.*, 2016 ▸), which features an anisotropic onset followed by an isotropic development. Xu and co-workers demonstrated that the redox pattern can be modulated by the arrangement of crystallographic orientations of the primary grains within a secondary NCM particle (Xu *et al.*, 2020 ▸). These particle-level observations, however, could be further complicated by the electrode-level micromorphology (Li *et al.*, 2022 ▸) and the effect of its distortion upon battery operation.

In Fig. 4 ▸(*e*), we present the final XANES maps of all five identified particles after the final charging. These particles exhibit different levels of inhomogeneity. Their XANES maps are even more distinct. Particles 1 and 5 clearly demonstrate an oxidized particle core and there is a significant offset from the particle center. Particles 2 and 3 both demonstrate a shrinking-core pattern, which is widely anticipated. Particle 4, on the other hand, exhibits a lower Ni oxidation state, which indicates that it could be partially deactivated, possibly due to a loss of contact. From the perspective of electrode deformation, we choose particle 3 as our spatial reference and illustrate the spatial movements of the other particles. Out of all the particles, particle 2 exhibits a distinct motion pattern that drifts slowly towards the upper right. The other particles demonstrate more random motion patterns with smaller amplitudes. In the data presented here, a correlation between electrode deformation and particle redox heterogeneity is not obvious. To understand this properly, many more experimental observations are needed and a high-throughput data curation pipeline with good automation is indispensable. The development presented in this work could potentially facilitate a systematic investigation of the correlation between a particle’s redox heterogeneity and the electrode-level non-rigid deformation, which can mutually modulate each other and collectively affect the cell performance.

## 5. Conclusion

We have proposed an efficient non-rigid alignment method for XANES reconstruction of battery NCM particles, enabling simultaneous tracking of multiple particles. This method captures the offset velocity, acceleration and distance of the particles, thereby facilitating an in-depth analysis of the coupling mechanisms involving morphology, compositional distribution and redox behavior. The significance of this method consists in its contribution to elucidating the coupling mechanism governing morphology, compositional distribution and redox heterogeneity, with profound implications for the design of next-generation batteries. Beyond its application to the battery NCM particles delineated in this study, our proposed method demonstrates considerable potential for examining chemical–physical coupling effects in diverse disciplines.

The sample recognition capabilities and accuracy of the deep learning model of the proposed method depend on the diversity and quality inherent in the training datasets. The principal limitations and prospective challenges intrinsic to this method include variations in morphology (size, thickness, radiation deformation and other factors), the X-ray absorbability of the samples, multiple scenarios of dynamic changes in the background, and the quality of the XANES projection images (multiple signal-to-noise ratio conditions). The extant training dataset encompasses a battery NCM particle sample acquired through TXM, supplemented by additional morphological samples featuring tungsten needles and specimens distinguished by the particle features made of gold. The applicability and precision of the model may encounter constraints when deployed in dissimilar domains characterized by substantial disparities in morphology and background features compared to those encompassed in the training datasets. However, this limitation is mitigated by the incorporation of unsupervised deep learning models in this work, addressing challenges associated with dataset expansion.

In response to evolving research efforts, future investigations could extend the diversity and quality of training data. This iterative process may involve optimizing the deep learning network model, thereby enhancing the method’s robustness and efficiency across various application scenarios spanning multiple disciplines and industries.

## Supplementary Material

Video S1. DOI: 10.1107/S1600577524000146/mo5272sup1.mp4

Video S2. DOI: 10.1107/S1600577524000146/mo5272sup2.mp4

Supporting Sections S1 and S2. DOI: 10.1107/S1600577524000146/mo5272sup3.pdf
