# Supplementary material for: Image registration for in situ X-ray nano-imaging of a composite battery cathode with deformation
Source: J Synchrotron Radiat. 2024 Feb 1;31(Pt 2):328–35. doi: 10.1107/S1600577524000146 (PMC10914177; doi:10.1107/S1600577524000146)
Supplement: Supplementary file 3 [file s-31-00328-sup3.pdf]

JOURNAL OF SYNCHROTRON RADIATION

Volume 31 (2024)

Supporting information for article:

Image registration for *in-situ* X-ray nano-imaging of composite battery cathode with deformation

Bo Su, Guannan Qian, Ruoyang Gao, Fen Tao, Ling Zhang, Guohao Du, Biao Deng, Piero Pianetta and Yijin Liu

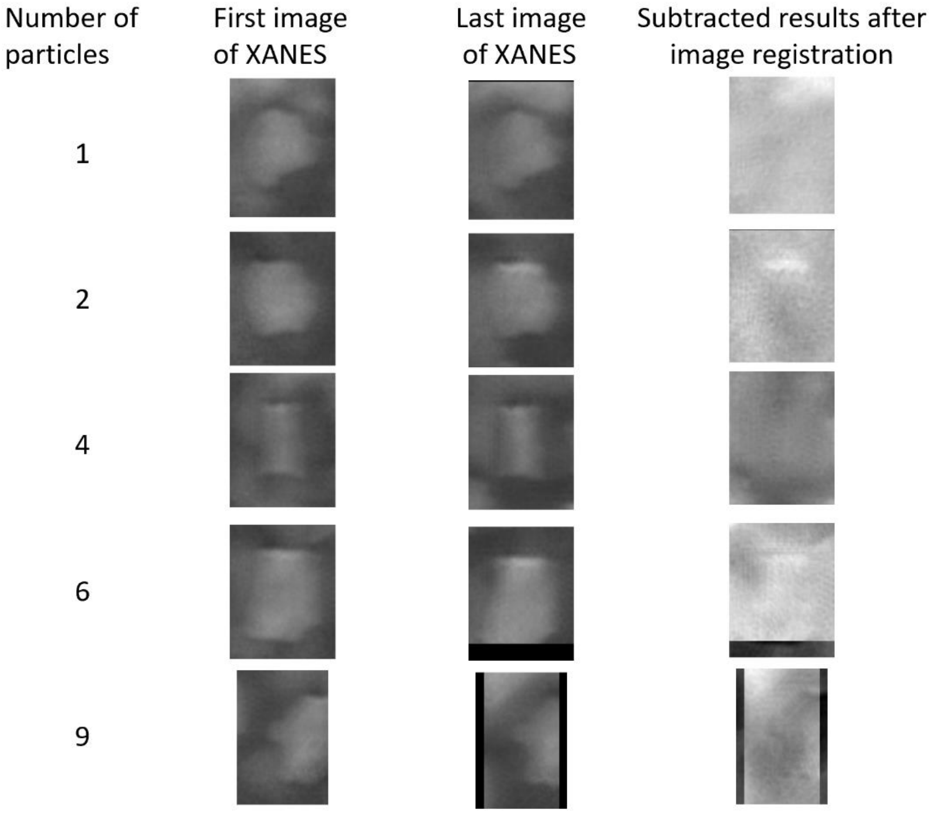

Figure S1 Analysis of the image alignment results of a XANES dataset. Supplementary results of some particles XANES data after alignment using our proposed method. The second and third columns of Figure S1 show the first (low-energy projection image) and the last (high-energy projection image) of these particles in the XANES data, respectively, and the results of the subtraction of these two projection images are shown in the fourth column, and the offset of these particles can be viewed in video1. Based on the results of the subtraction process depicted in Figure S1, it is evident that the contours of individual particles present in both the first and last projection images exhibit a substantial overlap following alignment. Moreover, any residual discrepancies in the overlapping areas can be attributed to the differential characteristics between high-energy and low-energy scans, which is an inherent aspect of the X-ray absorption near edge structure (XANES) technique, and does not impact the alignment of the images.

S1. Training and performance

These additional details serve as a supplement to Section 3.2, providing a comprehensive explanation of the training process and the impact of different training parameters on the dataset.

Firstly, the CycleGAN, is employed as an executor for coarse segmentation of the region of interest. It suffices to roughly identify the area where individual particles are located. The training approach aligns entirely with the conventional CycleGAN with its original training method (Zhu et al., 2017). The performance of this network can be referenced in the mentioned literature (Zhu et al., 2017), which provides various cases for consideration. In this work, we annotated 286 projection images (not necessarily one-to-one pairs) and completed training after 500 iterations.

Next, our primary focus is on the detailed extraction of image registration features using the Dual U-net model we employed. The training process, performance, and validation results for the Dual U-net are extensively described in the reference (Su et al., 2022), which is the basic part of our method presented in this paper. This model utilized a sample of a needle tip to better assess the needle offset by pixels and included the training process for the components of the Dual U-net and the validation results under different training parameters. Exemplified the training loss for each training epoch along with the associated valid deep learning network feature detecting performance. Corresponding validation results are appropriately labeled. In order to ascertain the experiment's validity, the training parameters were systematically tested at intervals of 100, 300, 500, 800, 1,000, 3,000, 5,000, 8,000, 10,000, 12,000, and 15,000.

We determined that a model with the highest number of training epochs produced feature maps most resembling the ground truth. Validation results showed models with over 500 epochs accurately generated feature maps for input projections but with introducing background noises. After 8,000 epochs, overfitting negatively impacted accuracy, but exhibited reduced background noise. Thus, we used two models with low and high training counts to form the Dual Unet model used for feature fine extraction. Compared to conventional template/feature alignment methods, our method reduces the alignment error rate, by an order of magnitude (Su et al., 2022).

S2. Data Enhancement

To improve the robustness of the proposed XANES projection image alignment method, we performed data enhancement on the training data, including random relative shifts of 10% to 30% horizontally and vertically, rotations of 2% to 30%, and the addition of 10%, 20%, and 30% random noise to the projected images. Moreover, we also used Cycle-GAN to artificially and randomly vary the position of cell NCM particles in the image, and the speed of its offset, for increasing the difficulty of non-rigid alignment.

References

Zhu, J.-Y., Park, T., Isola, P. & Efros, A. A. (2017). *Proceedings of the IEEE International Conference on Computer Vision*, pp. 2223-2232.

Su, B., Gao, R., Tao, F., Zhang, L., Du, G., Li, Z., Deng, B. & Xiao, T. (2022). *Nuclear Instruments and Methods in Physics Research Section A: Accelerators, Spectrometers, Detectors and Associated Equipment*, **1040**, 167242.
